# Comparison of continuously acquired resting state and extracted analogues from active tasks

**DOI:** 10.1002/hbm.22897

**Published:** 2015-07-15

**Authors:** Sebastian Ganger, Andreas Hahn, Martin Küblböck, Georg S. Kranz, Marie Spies, Thomas Vanicek, René Seiger, Ronald Sladky, Christian Windischberger, Siegfried Kasper, Rupert Lanzenberger

**Affiliations:** ^1^ Department of Psychiatry and Psychotherapy Medical University of Vienna Vienna Austria; ^2^ MR Center of Excellence, Center for Medical Physics and Biomedical Engineering, Medical University of Vienna Vienna Austria

**Keywords:** brain network, resting‐state fMRI, task regression, task‐derived resting state

## Abstract

Functional connectivity analysis of brain networks has become an important tool for investigation of human brain function. Although functional connectivity computations are usually based on resting‐state data, the application to task‐specific fMRI has received growing attention. Three major methods for extraction of resting‐state data from task‐related signal have been proposed (1) usage of unmanipulated task data for functional connectivity; (2) regression against task effects, subsequently using the residuals; and (3) concatenation of baseline blocks located in‐between task blocks. Despite widespread application in current research, consensus on which method best resembles resting‐state seems to be missing. We, therefore, evaluated these techniques in a sample of 26 healthy controls measured at 7 Tesla. In addition to continuous resting‐state, two different task paradigms were assessed (emotion discrimination and right finger‐tapping) and five well‐described networks were analyzed (default mode, thalamus, cuneus, sensorimotor, and auditory). Investigating the similarity to continuous resting‐state (Dice, Intraclass correlation coefficient (ICC), *R* ^2^) showed that regression against task effects yields functional connectivity networks most alike to resting‐state. However, all methods exhibited significant differences when compared to continuous resting‐state and similarity metrics were lower than test‐retest of two resting‐state scans. Omitting global signal regression did not change these findings. Visually, the networks are highly similar, but through further investigation marked differences can be found. Therefore, our data does not support referring to resting‐state when extracting signals from task designs, although functional connectivity computed from task‐specific data may indeed yield interesting information. *Hum Brain Mapp 36:4053–4063, 2015*. © 2015 The Authors Human Brain Mapping Published by Wiley Periodicals, Inc.

## INTRODUCTION

The neuroimaging community has increasingly focused on resting‐state and functional connectivity research. This trend is reflected by large‐scale databases like the 1,000 Functional Connectomes Project [Biswal et al., 2010], where a great number of resting‐state datasets are accumulated. These efforts have led to the identification of a multitude of well‐described networks, which are highly reproducible across different methods [Beckmann, 2005; Tomasi et al., 2009]. More importantly, numerous studies have been performed to assess differences in networks during resting conditions in various clinical populations [Broyd et al., 2009; Fox and Greicius, 2010]. This has revealed major insights on alterations in network organization in various psychiatric and neurological disorders such as major depression [Greicius et al., 2007], anxiety disorders [Hahn et al., 2011], and Alzheimer's disease [Sorg et al., 2007].

As a consequence, research interest for connectivity and changes in network parameters during task processing [Greicius, 2004] has also emerged. While relating resting‐state to task processing, Smith et al. [2009] have demonstrated that resting‐state networks are also connected during task engagement. Moreover, significant fractions of the trial‐to‐trial variance in task paradigms can be explained by coherent spontaneous activity, suggesting that ongoing intrinsic neuronal activity has an influence on behavior [Fox and Raichle, 2007]. It further has been hypothesized that ongoing activity in the brain establishes a functional architecture, in which neural responses are embedded but which cannot be fully isolated from spontaneous fluctuations [Sadaghiani et al., 2010].

These observations provide a strong argument for extraction of signals similar to resting‐state from task‐specific data. Methods applied in current research include the use of task data processed equal to resting‐state [Greicius, 2004; Jones et al., 2010; McFadden et al., 2014; Gimènez et al., 2012; Wu et al., 2011], concatenating baseline conditions from block‐designs [Fair, 2007; Fernandez‐Espejo et al., 2010; Honey, 2002; Kraus et al., 2014; Tomasi et al., 2009, 2010; Washington et al., 2013] as well as using residuals after regression against the task [Fair, 2007; Jones et al., 2010; Noonan et al., 2009; Shih et al., 2010; Weisberg et al., 2014]. Interestingly, there seems to be a trend in the literature to use the regression method [Korgaonkar et al., 2014; Muller et al., 2011], though other groups have recommended the use of baseline blocks [Fair, 2007; Fleisher et al., 2009; Hedden et al., 2009]. Generally, interpretation of these methods is still a matter of debate [Fair, 2007; Loitfelder, 2014; Muller et al., 2011; Sadaghiani et al., 2010]. The importance of appropriate methodological assessment has been recently highlighted in autism spectrum disorder [Muller et al., 2011]. In this case, differences in functional connectivity extracted from task designs could not be attributed unambiguously to either underlying pathophysiology or methodological differences across populations.

To sum up, extraction of resting‐state data from task‐designs indeed represents a highly relevant method in current fMRI research. However, there is a lack of consensus which technique performs best as comprehensive comparisons are missing. Application of a method that allows for extraction of signal similar to resting‐state from tasks would allow for utilization of previously acquired data, particularly in cases in which resting state paradigms were not acquired. Further, a method of this kind could illuminate details about the relationships between task and rest‐conditions.

To address this issue, we aimed to provide a thorough comparison of the applied techniques and to test potential methodological improvements. More precisely, we assessed which method best resembles continuous resting‐state networks. Two task paradigms (emotion discrimination and right finger‐tapping) as well as five different networks were compared. Furthermore, we modified the concatenation method [Fair, 2007] to account for subject‐specific shifts in delay of the hemodynamic response function (HRF), allowing us to avoid conservative cutting intervals.

## METHODS

### Participants

Twenty‐six healthy subjects (13 female), aged 25.6 ± 5.83 years (mean ± standard deviation) were included in the study. For the test‐retest comparison data from 20 subjects, aged 26.7 ± 5.91 years, partly overlapping with the above group has been analyzed. The second scan has been conducted one month after the first measurement. All subjects underwent standard medical examination and completed the Structural Clinical Interview for DSM‐IV (SCID) disorders, which was utilized to exclude persons suffering from neurological or psychiatric disorders. In addition, exclusion criteria included past substance abuse, intake of psychotropic medication, pregnancy, hormone treatment and contraindications for magnetic resonance imaging. All subjects provided written informed consent after detailed explanation of the study protocol by an experienced psychiatrist and were reimbursed for their participation. The study was approved by the Medical University of Vienna ethics committee and procedures were conducted according to the Declaration of Helsinki.

### fMRI Scanning

All participants underwent fMRI scanning in a Siemens Magnetom 7T MR scanner (EPI‐sequence: TE = 23 ms, TR = 1.4 s, flip angle = 62°) located at the Medical University of Vienna as described previously [Hahn et al., 2013]. Each subject completed one continuous resting‐state scan as well as two paradigms in a typical block design. The acquired images comprised voxel dimensions of 1.48 mm × 1.48 mm × 2 mm (+1 mm slice gap), and dimensionality of 128 × 128 voxels, comprising 32 slices. All stimuli and timings were programmed in the cogent toolbox for MATLAB and were presented to the subjects by a projector.

### Resting State

During the resting‐state acquisition (total duration = 360 s) subjects have been instructed to think about nothing in particular, while simply viewing the cross‐hair, that is, stay relaxed but awake in the scanner with eyes open [Hahn et al., 2011; Weissenbacher, 2009].

### Functional Paradigms

The first paradigm was the right‐finger‐tapping (RFT) task. Here, the subject was instructed to alternately tap the index and middle finger towards the thumb during a stimulus‐phase in which flickering checkerboards were shown (8 Hz). The RFT consisted of 6 × 10 s tapping periods, and 7 × 10 s baseline‐blocks (total paradigm length = 130 s). During the baseline‐blocks, which are of main interest in this work, a crosshair was presented to the subjects. The participants were instructed that the baseline blocks represent a short pause between the trials. The description of the crosshair sequence was the same for the emotion‐discrimination task. This task was chosen, due to its simplicity, as the task is well understood and has distinct areas of activation [Biswal et al., 1995, 2010]. Furthermore, the paradigm has found widespread application with good reproducibility [Gountouna et al., 2010].

The emotion discrimination task (EDT) consisted of 4 blocks of emotion‐discrimination and 4 blocks of object‐discrimination with 20 s each, alternated by 8 ×20 s baseline‐blocks, plus one baseline‐block at the end (total duration = 320 s). This task‐design is an established paradigm [Hariri, 2002] and is more sophisticated in comparison to the finger‐tapping task, as it involves emotional facial stimuli. This task was chosen since it represents a widely applied paradigm to elicit emotional processing. Furthermore, the duration of baseline blocks, as well as total paradigm length were longer as compared to the RFT‐task.

### Data Preprocessing

Preprocessing of the data, unrelated to the method of extraction or paradigm, was carried out in SPM8 using default parameters unless otherwise specified. This included slice timing correction (reference=first slice) [Sladky et al., 2011], realignment (reference=mean image), spatial normalization to a scanner‐specific EPI template in MNI‐space [Hahn et al., 2013] as well as spatial smoothing using an 8 mm × 8 mm × 8 mm FWHM Gaussian kernel. Following preprocessing, the next step was to test the different methods to extract resting‐state from task‐specific data and/or for removing task‐related signals (see below).

Multiple linear regression was applied to remove movement parameters as calculated by SPM, as well as white‐matter, ventricular and global signal, followed by band‐pass filtering (0.009 < *f* < 0.08Hz). Finally, cross‐correlation to seed regions was calculated and resulting maps were transformed to z‐scores for group comparison [Weissenbacher, 2009]. To acknowledge the ongoing dispute about global signal regression (GSR), we also performed the calculations for one task dataset without applying GSR.

### Extraction of Resting‐State From fMRI Task‐Data

The following 4 approaches of resting‐state extraction from task‐specific data have been applied. The actual resting‐state data was used for comparison, where the duration was adapted to fit the duration after task removal.

#### Method 1: Original unmodified data (ORIG)

In this method, functional connectivity was calculated from unmodified task data in the same way as it was done for resting‐state data [Greicius, 2004]. As previously stated, the task is either right finger‐tapping (RFT) or an EDT. Besides the filtering related to the seed‐based correlation (i.e., band‐pass and regression against “physiological noise” including white matter, ventricular and global signal) [Weissenbacher, 2009] no efforts were done to remove the signal that is associated with the task [Greicius, 2004].

#### Method 2a: Baseline block extraction (BLOCK)

Resting‐state blocks were extracted from the design and concatenated as described previously [Fair, 2007]. After the end of each task block, a fixed duration of ∼15 s (dependent on rounding of TR) was removed, allowing the hemodynamic response to return to a baseline state. At the start of each task‐block, an interval of 5 s was assumed to be the resting‐condition. This method, when applied in the right finger‐tapping paradigm, resulted in extracted resting‐state durations lower then 30 s, as the cutting intervals are conservative, generously avoiding possible contaminations. We, therefore, excluded method 2a for the finger‐tapping paradigm due to insufficient amount of data points.

#### Method 2b: Baseline block extraction with variable shift (BLOCKvar)

Similar to method 2a, baseline blocks were extracted from the task‐design and concatenated to create a resting‐state analogue. Additionally, the delay of the HRF was estimated for each subject individually in order to eliminate potential task influences. Instead of using a standard HRF with fixed delay (e.g., from SPM), we aimed to estimate the delay individually for each subject, and then adjust the cutting intervals. This was done because the hemodynamic response varies across subjects and even sessions [Aguirre et al., 1998; Cunnington et al., 2003; Handwerker, 2004]. Hence, individual estimates of the HRF shift should provide more accurate representations in contrast to one static model for the entire group. We assumed that shortly after the peak, further changes in response would be minimal. We also assumed that the actual baseline condition starts at the onset of the cross‐hair plus the shift, lasting the same duration as the presentation of the crosshair.

Response time‐to‐peak was estimated as follows: First a spherical region of interest (5 mm diameter) was placed in the occipital cortex bilaterally (center at *x,y,z* = +17 mm/−22 mm, −90 mm, +2mm MNI‐space) Next, we created a theoretical boxcar‐model for each subject, given by onsets and durations of the block design as extracted from the scanning log‐files.

To identify the individual HRF shift, the modeled box‐car was shifted in time by 1 TR‐steps (i.e., 1.4s steps = 1TR), while analyzing the correlation with the measured response. This enabled us to search for the maximum correlation, which was assumed to represent the best estimate for the individual shift. This shift was limited to a maximum of 7 times the TR (7 × 1.4s = 9.8s), based on previous estimates of the shape of the HRF [Handwerker, 2004]. Individual HRF delays as computed in the BLOCKvar method were 5.2 s ± 1.32 s (mean ± sd).

#### Method 3: Residual time course after regression (REG)

In this method we tested if multi‐linear regression enables an effective removal of task effects from the BOLD signal [Fair, 2007]. Here, the same theoretical boxcar‐model as in method 2b was used. After convolution with the HRF model from SPM8, the estimated responses were used for the multilinear regression. The residuals from this regression are assumed as time course with removed task effects [Fair, 2007; Korgaonkar et al., 2014].

#### Combination of method 2b+3: Block extraction after regression (BlockREG)

For this technique, regression and block extraction have been combined. First, regression was performed as described in method 3 (REG). After the task removal from REG, we concatenated the baseline‐blocks from the residuals, similar to method 2b.

### Resting‐State Networks

**Figure 1:**
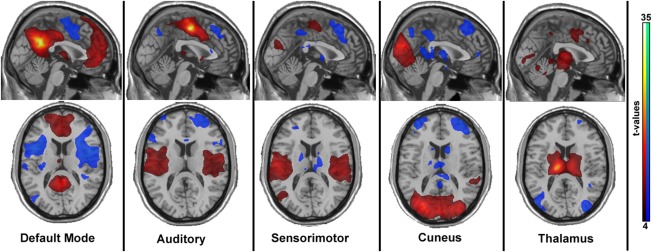
The networks used for analysis, represented via one‐sample *t*‐tests as conducted with SPM. The maps are based on the individual z‐score maps of the first resting‐state measurement of the test‐retest measurements (*n*=20). Maps represent *t*‐values, thresholded between 4 and 35. Although the auditory and sensorimotor networks look similar, one seed was placed in the motor cortex (“auditory”), and the second in the temporal gyrus (“sensorimotor”) as published previously [Smith et al., 2009]. [Color figure can be viewed in the online issue, which is available at http://wileyonlinelibrary.com.]

Three coordinates for the seed of the resting‐state networks were taken from Tomasi et al 2011. In particular, the chosen networks correspond to the “default mode”‐ (*x* = 4 mm, *y* = −52 mm, *z* = 29 mm), the “cuneus”‐ (*x* = −24 mm, *y* = −80 mm, *z* = 18 mm) and the “thalamus”‐network (*x* = −12 mm, *y* = −19 mm, *z* = 8 mm). Additional two networks have been chosen from an independent component analyses based approach [Smith et al., 2009]. These are defined as the peaks from resting state network “sensorimotor” (*x* = 0 mm, *y* = −12 mm, *z* = 50 mm) and “auditory” (*x* = −60 mm, *y* = −4 mm, *z* = 2 mm). Networks are displayed in figure 1.

To account for potential outliers and to decrease influences of noise, the seed was defined as a 3×3×3 voxel cube around the coordinate instead of using a single voxel [Cole et al., 2010]. The average time course was then extracted and cross‐correlated with the entire brain, followed by z‐transformation (see data preprocessing section).

### Statistical Testing

**Figure 2:**
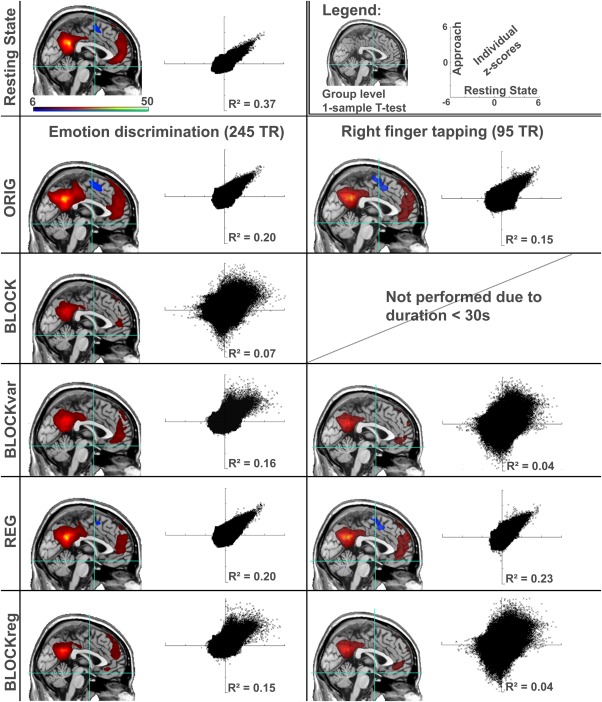
Comparison between continuous and extracted resting‐state connectivity for the default mode network. Maps represent one‐sample *t*‐tests across the entire group for each method. In the scatter plots, resting state (*x*‐axis) and the according method of extraction (*y*‐axis) are compared for all brain voxels across all individual *z*‐score maps. Hence, the top left scatterplot represent test‐retest evaluation of two resting‐state scans. Scaling is uniform for all images (*t*‐values from 6.09–50, *P*<0.05 FWE‐corrected), and scatter‐plots (−6 to +6). For the finger‐tapping the BLOCK method is not shown, as the resulting signal comprised less then 30 s, and therefore considered too short. Visually, the networks are highly similar, but through further investigation marked differences can be found. [Color figure can be viewed in the online issue, which is available at http://wileyonlinelibrary.com.]

One sample *t*‐tests of the DMN were computed with SPM for visualization purposes (Fig. 2). The DMN has been chosen for this visualization as it has the highest reliability as indicated by all three similarity measures (Tables 1, 2, 3).

**Table 1:** Intraclass correlation coefficient between maps derived from method of extraction and continuous resting‐state

	Cuneus	Auditory	Sensorimotor	Default Mode	Thalamus
EDT (with GSR)					
BLOCKreg	0.26^a^	0.29^a^	0.25^a^	0.34^a^	0.13^a^
BLOCKvar	0.28^a^	0.30^a^	0.24^a^	0.36^a^	0.13^a^
BLOCK	0.17^a^	0.19^a^	0.13^a^	0.20^a^	0.13^a^
REG	**0.29** a	**0.34**	**0.29** a	0.41^a^	**0.20** a
ORIG	0.32^a^	0.32^a^	0.26^a^	**0.42** a	0.18^a^
RFT (with GSR)					
BLOCKreg	0.07^a^	0.10^a^	0.08^a^	0.17^a^	0.06^a^
BLOCKvar	0.07^a^	0.09^a^	0.08^a^	0.17^a^	0.05^a^
REG	**0.27** a	**0.22** a	**0.18** a	**0.43** a	**0.16** a
ORIG	0.21^a^	**0.22** a	0.17^a^	0.36^a^	0.15^a^
Test‐Retest (with GSR)	0.51	0.41	0.44	0.58	0.36
EDT (without GSR)					
BLOCKreg	0.41^a^	0.45^a^	0.40^a^	0.41^a^	0.39^a^
BLOCKvar	0.42^a^	0.47	0.45^a^	0.42^a^	0.42^a^
BLOCK	0.30^a^	0.32^a^	0.30^a^	0.23^a^	0.37^a^
REG	**0.48** a	**0.52**	0.49^a^	**0.46**	0.43^a^
ORIG	0.49^a^	0.50	**0.51** a	**0.46** a	**0.44** a
Test‐Retest (without GSR)	0.65	0.59	0.66	0.57	0.57

a It denotes network significantly different to test‐retest, where extraction methods with and without GSR are compared to the corresponding test‐retest with and without GSR, respectively (*P* < 0.05 *post hoc t‐*tests).

Values represent the average across all subjects, higher ICC indicates better overlap between methods. Highest values for each network are marked in bold. Test‐retest values between two resting‐state scans are presented for comparison

**Table 2:** Average *R* ^2^ values between maps derived from method of extraction and actual resting‐state

	Cuneus	Auditory	Sensorimotor	Default Mode	Thalamus
EDT (with GSR)					
BLOCKreg	0.12^a^	0.13^a^	0.11^a^	0.15^a^	0.05^a^
BLOCKvar	**0.13** a	0.14^a^	0.10^a^	0.16^a^	0.05^a^
REG	0.11^a^	**0.16**	**0.13** a	**0.20**	**0.08** a
BLOCK	0.06^a^	0.07^a^	0.04^a^	0.07^a^	0.04^a^
ORIG	**0.13** a	0.13^a^	0.11^a^	**0.20** a	0.07^a^
RFT (with GSR)					
BLOCKreg	0.03^a^	0.03^a^	0.02^a^	0.04^a^	0.02^a^
BLOCKvar	0.03^a^	0.03^a^	0.02^a^	0.04^a^	0.02^a^
REG	**0.10** a	**0.09** a	**0.06** a	**0.23** a	**0.05** a
ORIG	0.07^a^	**0.09** a	**0.06** a	0.15^a^	**0.05** a
Test‐Retest (with GSR)	0.30	0.20	0.24	0.37	0.17
EDT (without GSR)					
BLOCKreg	0.41^a^	0.46^a^	0.41^a^	0.41^a^	0.39^a^
BLOCKvar	0.43^a^	0.47^a^	0.45^a^	0.42^a^	0.42^a^
REG	0.31^a^	0.32^a^	0.30^a^	0.26^a^	0.37^a^
BLOCK	0.53^a^	**0.56**	0.54^a^	**0.49** a	**0.47** a
ORIG	**0.54** a	0.53^a^	**0.56** a	0.48^a^	**0.47** a
Test‐Retest (without GSR)	0.69	0.65	0.71	0.61	0.63

a It denotes network significantly different to test‐retest, where extraction methods with and without GSR are compared to the corresponding test‐retest with and without GSR, respectively (*P* < 0.05 *post hoc t*‐tests).

Values represent the averaged across all subjects; higher value indicates better overlap between methods. Highest values for each network are marked in bold. Test‐retest values between two resting‐state scans are shown for comparison.

**Table 3:** Dice similarity metric between maps derived from method of extraction and actual resting‐state

	Cuneus	Auditory	Sensorimotor	Default Mode	Thalamus
EDT (with GSR)					
BLOCKreg	0.35^a^	0.36	**0.35**	0.39^a^	0.27
BLOCKvar	0.36^a^	0.36	0.34^a^	0.40^a^	0.27
BLOCK	0.34^a^	0.35	0.32^a^	0.35^a^	**0.30**
REG	0.35^a^	0.36	0.34^a^	0.40^a^	0.28
ORIG	**0.37** a	**0.37**	0.34^a^	**0.43** a	0.27
RFT (with GSR)					
BLOCKreg	0.31^a^	**0.33** a	0.33 *	0.36^a^	**0.29**
BLOCKvar	0.30^a^	0.30^a^	**0.33** a	0.35^a^	**0.29**
REG	**0.35** a	**0.33** a	0.30^a^	**0.41** a	0.25^a^
ORIG	0.32^a^	0.32^a^	0.29^a^	0.40^a^	0.27
Test‐Retest (with GSR)	0.52	0.40	0.42	0.51	0.31
EDT (without GSR)					
BLOCKreg	0.54	0.56	0.51^a^	**0.49**	0.52
BLOCKvar	0.54	**0.57** a	0.51	0.48	0.53
BLOCK	0.48^a^	0.52	0.49^a^	0.43^a^	**0.56**
REG	**0.55**	**0.57** a	**0.52**	0.47	0.5
ORIG	**0.55**	0.54^a^	0.50	0.46	0.4
Test‐Retest (without GSR)	0.61	0.61	0.66	0.53	0.57

a It denotes network significantly different to test‐retest, where extraction methods with and without GSR are compared to the corresponding test‐retest with and without GSR, respectively (*P* < 0.05 post hoc *t*‐tests).

Highest values for each network are marked in bold, higher value indicates better overlap between methods. Test‐Retest between two resting‐state scans values are shown for comparison

ICC [McGraw and Wong, 1996] and Dice similarity measure [Birn et al., 2013] have been calculated for each method with continuous resting‐state as reference to identify similarities between both conditions, and to enable comparison with previously published literature. The ICC was estimated across all brain voxels using unthresholded individual z‐score maps [McGraw and Wong, 1996]. For the calculation of the Dice measure a binary threshold of 0.3 was applied as published previously [Birn et al., 2013]. Hence in contrast to the Dice metric, the ICC does not rely on a binary threshold. Both indices range from zero to one, where zero represents no similarity and one represents identical sets.

Of note, all comparisons (*t*‐tests, Dice, ICC, and *R* ^2^) were computed for the entire brain, masked to exclude areas outside the cortex, without restriction to a particular network. To assess differences in similarity metrics between a given method of extraction and continuous resting‐state, a mixed factorial ANOVA was implemented in SPSS, followed by *post hoc* two‐sample *t*‐tests. This was done for each metric (Dice, ICC, and *R* ^2^) as well as for both tasks (RFT, EDT). Here, correlation values have been z‐transformed beforehand to ensure normal distribution.

## RESULTS

Computing the DMN as an average across the entire study group resulted in functional connectivity maps which were visually comparable to actual resting‐state for each of the extraction methods (maps in Fig. 2). However, further investigation of the individual functional connectivity showed remarkable differences for these methods (scatterplots in Fig. 2). Here, the explained variability was markedly lower for the methods cutting out baseline blocks, than for those using the entire scan. A decrease in similarity was also observed for the shorter right finger tapping paradigm, compared to the longer emotional discrimination task. It should be noted once again that the duration of the resting‐state has been adjusted to fit the duration of the method with which it is compared, ensuring that the functional connectivity maps have been derived from data with the same amount of time‐points.

For the test‐retest data, values of ICC and Dice were in a comparable range as reported previously [Birn et al., 2013] (Tables 1 and 3). Importantly, the majority of extraction methods yielded significantly lower similarity metrics than test‐retest. Moreover, this was not consistent across the different networks assessed, that is, different approaches yielded variable similarity values for different networks. Again, methods using the entire scan were most alike to that of test‐retest, with REG showing highest similarity, followed by ORIG. It should be noted that R^2^ (Table 2), as well as the ICC seemed more stable compared to the Dice, which might be related to the binary threshold of the Dice measure. *R* ^2^ as well as ICC provided results consistent across different networks, which was not the case for the Dice metric.

For the mixed model ANOVA in methods utilizing GSR, significant differences were found for the ICC for main effect of method in EDT (*F*[5,26] = 26.7) and RFT (*F*[4,26] = 25.3), as well as for the *R* ^2^ in EDT (*F*[5,26] = 64.4) and RFT (*F*[4,26] = 69.9, all *P*<0.001). DICE coefficient was not significant for EDT (*F*[5,26] = 1.7, *P*=0.14) and RFT (*F*[4,26] = 0.264, *P*=0.85). *Post hoc* two‐sample *t*‐tests showed that all methods of resting‐state extraction gave significantly lower similarity values within the vast majority of networks (Tables 1, 2, 3).

In addition, we carried out the analysis also without GSR for the EDT task. Similar to the results with GSR, the mixed model ANOVA showed significant differences for the main effect of method in the ICC (*F*[5,26]=15.8) and the *R* ^2^ (*F*[5,26]=17.2, both *P*<0.001). In line with the calculation including GSR, the DICE coefficient (*F*[5,26]=0.7, *P*=0.56) was not significant.

## DISCUSSION

This work aims to provide a thorough assessment of various methods used to gather resting‐state signal from task‐specific fMRI paradigms. In comparison to previous studies, we add important information by assessing similarity across different methods, whereas a strong focus on nonsignificant differences has prevailed before. The evaluation across different networks and paradigms indicated that using the residuals from a regression against task‐effects (REG) was most similar to resting‐state data, although still significantly different.

Concerning similarity, REG (regression against task effects) as well as ORIG (unmanipulated task data) showed the highest values for all metrics. One could argue that this is influenced by the final duration of the data after correcting for task‐effects. Methods providing shorter durations after extensive removal of blocks yielded lower similarity scores. Due to fewer samples, this means that the variance of the estimated effect, and therefore, uncertainty is greater for the methods having a lower amount of samples in the time‐domain. This is a disadvantage in terms of interpretability of data and an argument for using regression. When using REG, the output is the longest in terms of duration, which results in increased stability of the results. This is an advantage for REG as the duration of resting‐state scans is a topic of debate in which the general assumption is “the more the merrier” [Birn et al., 2013; Murphy et al., 2007] Murphy et al. also highlight an additional advantage of REG, which is not concatenating data, therefore, avoiding adverse effects concerning the signal‐to‐noise ratio. If one assumes continuous intrinsic activity [Sadaghiani et al., 2010] that should be investigated, repeatedly introducing sudden changes (i.e., high frequency components) by concatenation of time courses in the continuum might substantially alter the underlying signal properties, and the frequency spectrum.

Implementing an individual estimation of the HRF shift (BLOCKvar), we aimed to improve the previously introduced method of extracting resting‐state signal from baseline blocks (BLOCK) [Fair, 2007]. This allowed a less conservative cutting interval resulting in longer scan duration after task‐effect removal, which indeed improved the method. Furthermore, influences from task onto the baseline block seemed to be negligible after a delay of a few seconds. However, all methods cutting out baseline blocks consistently showed lower similarities to resting‐state than regressing out task effects.

Interestingly, one can easily reproduce “resting‐state” maps even when using unmodified data acquired during paradigms (Fig. 2). This is inherent to the DMN's definition as “a network that is routinely deactivated during task states” [Raichle, 2000], and is in accordance with the previously discussed assumption of continuous intrinsic activity [Sadaghiani et al., 2010; Smith et al., 2009]. These networks do accommodate a certain amount of coactivation and spatial consistency represented via correlation, also during task‐states [Greicius, 2004]. However, it seems that this issue is not always taken into account when interpreting results of such analyses. Computing functional connectivity networks from task data may indeed yield interesting findings, but one must be aware of the fact that the resulting networks are statistically different to those calculated from resting‐state data, although visually similar. In Figure 2, all networks seem highly similar, and share an enormous overlap, due to the consistent relations between parts of the network during rest and task [Sadaghiani et al., 2010; Smith et al., 2009]. However, investigating the individual connectivity instead of group‐averaged networks differences between the methods are evident. This is particularly important for clinical applications [Loitfelder, 2014; Muller et al., 2011], where individual functional connectivity values may be related to treatment response. However, our findings demonstrate that it is exactly this individual variation, which becomes unstable, especially when cutting out baseline blocks and to a lesser but still significant extent when regressing out task‐signals.

Some studies report findings to be identical to those acquired from resting‐state data [Greicius, 2004; Korgaonkar et al., 2014], while others observed overt differences even in unpaired study designs [Fair, 2007]. Although visual comparison seems appealing, misleading conclusions may be drawn. The voxels exhibiting significant connectivity can easily vary, especially in the outermost area of the network, and for smaller sample sizes [Damoiseaux, 2006]. Therefore, an unbiased quantitative assessment of network overlap is desirable. Here, we used the ICC and Dice similarity metrics [Birn et al., 2013], next to *R* ^2^. The observation that ICC showed more consistent results than the Dice might be related to the different computation of these two values. Dice is computed from binary images after choosing an arbitrary threshold (here: 0.3 for comparison with previous reports [Birn et al., 2013]), whereas ICC as well as *R* ^2^ are computed from connectivity maps, without thresholding. This inconsistency may also explain the nonsignificant F‐test when comparing the Dice metric across different methods.

Considering the differences between resting‐state analysis including GSR and the one omitting GSR the gain in the individual metrics achieved by excluding GSR is striking. In individual cases the test‐retest reliability for single networks is more than doubled as compared to the analysis including GSR. This effect is also consistently reflected in elevated test‐retest scores. This is in line with previous findings; however, test‐retest reliability alone does not allow conclusion of the validity of the data, as this could also be due to noise [Shirer, 2015]. Another interesting effect is that while the values for the default mode with GSR are in a range comparable to the results without GSR, the other networks seem to level up to numerically similar scores as the DMN. This is in line with the argumentation that different networks are differently related to the global signal, and therefore differently affected by GSR [Gotts, 2013]. Nevertheless, the different networks and methods were still significantly different from the test‐retest scores when omitting GSR, similar to the results with GSR. In other words, GSR did not change the finding, that extraction of resting‐state data from task‐related signals did indeed yield significant differences to continuously acquired resting‐state.

We want to further emphasize that a nonsignificant finding in a *t*‐test does not imply similarity, as often falsely concluded. Hence it is worth to mention that tests for significant differences (ANOVA, *t*‐tests, etc.) are valid to assess whether the null hypothesis (H_0_=no difference) can be rejected, and for that specific purpose only. Still, similarity has been concluded recently, where the default mode network did not show significant differences between actual resting‐state and residuals from tasks [Korgaonkar et al., 2014]. Interestingly, visual comparison showed even stronger differences in DMN connectivity within the medial prefrontal cortex [Korgaonkar et al., 2014] as compared to our study (Fig. 2), whereas we indeed observed significant differences. Our data indicates that differences to actual resting‐state still remain, regardless of the method of extraction and findings should be interpreted with care. We do not claim that results obtained by approaches extracting resting‐state from task‐specific data are not valid. However, considering the marked differences we would like to emphasize the importance of not referring to any of these methods as resting‐state.

## CONCLUSION

To summarize, all of the described methods available for the extraction of resting‐state data from task‐specific fMRI designs are actively applied and relevant in current research. However, the assessment demonstrated substantial differences in comparison to continuous resting‐state. Although regression against task‐effects (REG) was the method showing highest similarity with the original resting state, the resulting functional connectivity maps were still significantly different. Considering this mismatch with continuous resting‐state, results obtained from extracted signals should be interpreted with care, and not be referred to as resting‐state.
